# Supplementary material for: Knocking out TMEM38B in human foetal osteoblasts hFOB 1.19 by CRISPR/Cas9: A model for recessive OI type XIV
Source: PLoS One. 2021 Sep 28;16(9):e0257254. doi: 10.1371/journal.pone.0257254 (PMC8478202; doi:10.1371/journal.pone.0257254)
Supplement: S1 Materials and methods — (DOCX) [file pone.0257254.s001.docx]

**S1 Supporting Materials and Methods**

***Knock out in vitro model generation***

Three target sequences on TMEM38B (NG_032971.1), one on exon 2 (5’- GCTATGCTCCACTGTTTTGGTGG -3’, nt 16120-16142) and two on exon 3 (5’-AGAACTTGGAAAATAGTAGG-3’, nt 32107-32126 and 5’–TGGATAGTCATGATAGCTAT-3’, nt 32165-32186) were selected using the bioinformatic tools CHOPCHOP [1] and CRISPRscan [2, 3]. The target regions were sequenced and the three RNA guides (gRNA) were synthetized by annealing the two synthetic complementary oligonucleotides which were mixed in equimolar amount (100 µM) and annealed with the following cycle: 3 min at 95 °C followed by 1 min at 25 °C. The expression plasmids psCas9(BB)-2A-GFP (Addgene, PX458) and pSpCas9(BB)-2A-PURO (Addgene, PX459), which contains the Cas9 sequence under control of β-actin promoter [4], were used for the cloning of the double strand fragment of the RNA guides following linearization with BbsI enzyme (New England Bi-oLabs, Ipswich, Massachusetts, USA). To verify the correctness of the insert the clones were sequence (GATC Biotech AG). gRNAs were obtained after plasmid linearization with BamHI (Promega, Fitchburg, Wisconsin, USA) and purification with Nucleospin Gel and PCR Clean-up Kit (Macherey-Nagel). Briefly, *in vitro* transcription was performed using the MEGAshortscript T7 Kit (Invitrogen, Vilnius, Lithuania), following manufacturer’s instruction. The gRNAs were purified using mirVana miRNA Isolation Kit (Invitrogen, Vilnius, Lithuania). gRNA quality and size were checked by electrophoresis on 10% (v/v) polyacrylamide-Urea-SDS gel.

***hFOB 1.19 transfection***

For hFOB 1.19 cells transfection 1x10^6^ cells/well were plated into 6 well/plate and cultured for 24 hours in growing medium. The transfection with pSpCas9(BB)-2A-GFP, pSpCas9(BB)-2A-GFP-gRNA-2, pSpCas9(BB)-2A-PURO, pSpCas9(BB)-2A-PURO-gRNA-2, pSpCas9(BB)-2A-PURO-gRNA-3.1 and pSpCas9(BB)-2A-PURO-gRNA-3.2 was performed using LTX DNA transfection reagent (Invitrogen, Carlsbad, California, USA) following manufacturer’s indication. Cells transfected using pSpCas9(BB)-2A-PURO, pSpCas9(BB)-2A-PURO-gRNA2, pSpCas9(BB)-2A-PURO-gRNA3.1 and pSpCas9(BB)-2A-PURO-gRNA3.2 were selected using 2 µg/ml puromycin added to the media for 48 hours. Transfection efficiency was evaluated by measuring the percentage of GFP^+^ cells on total plated cells 48 h post transfection. Then, the DNA was extracted, and the target specificity was evaluated using T7 endonuclease assay. Briefly, 10 µL of each PCR amplicon underwent a denaturing/annealing cycle: 5 min at 94 °C, followed by cooling to 85 °C, at -2 °C per sec and further to 25 °C, at -0.1 °C per sec. The annealed amplicon was digested with 0.2 U/mL T7 endonuclease I (New England BioLabs, Ipswich, Massachusetts, USA) at 37 °C for 1 h, then the sample was run on 8% (v/v) polyacrylamide gel. T7 only cuts heteroduplex amplicons. The targeting was confirmed by Sanger Sequencing. To obtain the single clonal lines, transfected cells were plated in 96 well plate at 2-8 cells/well density. Wells containing single cells were screened the day after plating. The untransfected hFOB cells and WT clones were used as control for the experiments, as indicated in the figure legends.

***Genotyping***

DNA was extracted following cells lysis in 100 mM Tris HCl pH 8.5, 5 mM EDTA, 0.2% SDS (w/v), 200 mM NaCl, 2.5 mg/mL proteinase K (Sigma Aldrich, Darmstadt, Germany) at 37 °C overnight, by isopropanol precipitation and resuspension in 20 mM Tris-HCl, 1mM EDTA, pH 8.0. To evaluate the targeting success, T7 Endonuclease assay was performed as described above. The genomic region surrounding the target se-quences was amplified using the following primers: forward 5'- ACTTTAC-CATTTCAGGAGCAGC-3' (16058-16079 nt) and reverse 5'-CACATAGGTCACCATACTGGC-3' (16237-16258 nt) primers for exon 2; and forward 5'-TGTGTTTTGCCAAATTGTTGTT-3' (31917-31938 nt) and reverse 5'-CAGCCAACAGAAACAAGGTTTT-3' (32323-32344 nt) primers for exon 3.

To genotype the targeted clonal lines, specific restriction enzymes were used after PCR amplification of the targeting region. Enzymes able to cut only the WT sequence were selected, for exon 2 BstXI and XcmI (New England Biolabs) and for exon 3 BspHI and BccI (New England Biolabs). The digestion products were checked on 8% (v/v) polyacrylamide gel. To determine the specific mutations inserted by the NHEJ repair system, the alleles were sequenced following subcloning in TOPO TA vector.

References

1. Labun K, Montague TG, Krause M, Torres Cleuren YN, Tjeldnes H, Valen E. CHOPCHOP v3: expanding the CRISPR web toolbox beyond genome editing. Nucleic Acids Res. 2019 07;47(W1):W171-W74.

2. Doench JG, Hartenian E, Graham DB, Tothova Z, Hegde M, Smith I, et al. Rational design of highly active sgRNAs for CRISPR-Cas9-mediated gene inactivation. Nat Biotechnol. 2014 Dec;32(12):1262-7.

3. Moreno-Mateos MA, Vejnar CE, Beaudoin JD, Fernandez JP, Mis EK, Khokha MK, et al. CRISPRscan: designing highly efficient sgRNAs for CRISPR-Cas9 targeting in vivo. Nat Methods. 2015 Oct;12(10):982-8.

4. Ran FA, Hsu PD, Wright J, Agarwala V, Scott DA, Zhang F. Genome engineering using the CRISPR-Cas9 system. Nat Protoc. 2013 Nov;8(11):2281-308.
