# Supplementary material for: Knocking out TMEM38B in human foetal osteoblasts hFOB 1.19 by CRISPR/Cas9: A model for recessive OI type XIV
Source: PLoS One. 2021 Sep 28;16(9):e0257254. doi: 10.1371/journal.pone.0257254 (PMC8478202; doi:10.1371/journal.pone.0257254)
Supplement: S1 Results — (DOCX) [file pone.0257254.s002.docx]

**S1 Supporting Results**

CRISPR/Cas9 TMEM38B gene targeting in hFOB

One of the gRNAs was chosen on exon 2 (gRNA-2), encoding for 2 of the 4 transmembrane domains of TRIC-B (TM1 and TM2) and two on exon 3 (gRNA-3.1 and -3.2). The hFOB DNA in the selected target region was first sequenced to confirm the absence of polymorphisms that could compromise guides base pairing and Cas9 cleavage. The guides were subcloned in the expression vectors pSpCas9-2A-GFP and pSpCas9-2A-PURO, in which the green fluorescent protein (GFP) and the puromycin resistance gene (PURO) were expressed, respectively, together with the Cas9 (**Figure 1A**). First, using the constructs pSpCas9(BB)-2A-GFP and pSpCas9(BB)-2A-GFP-gRNA-2, a 60-70% transfection efficiency and target specificity were demonstrated (**Supplementary Figure S1A, B**). Then, cells were transfected with pSpCas9(BB)-2A-PURO, pSpCas9(BB)-2A-PURO-gRNA2, pSpCas9(BB)-2A-PURO-gRNA3.1 and pSpCas9(BB)-2A-PURO-gRNA3.2. A short incubation in presence of the drug enhances the number of surviving transfected cells, facilitating the screening protocol. Following transfection, only the gRNA-2 and gRNA-3.2 specifically targeted *TMEM38B*, as evaluated by T7 endonuclease I (T7EI) assay (**Supplementary** **Figure S2A**). T7E1 and restriction endonuclease digestions were employed to discriminate among targeted and WT clones. In particular, two endonucleases recognizing the WT sequence in the region of the cut envisaged by Cas9 were chosen for each guide to optimize the detection of the mutations inserted by non-homologous-end-join (NHEJ) repair system (**Supplementary Figure S2, Supplementary Tables S1-2-3**).
