# Supplementary figures and images for: Knocking out TMEM38B in human foetal osteoblasts hFOB 1.19 by CRISPR/Cas9: A model for recessive OI type XIV
Source: PLoS One. 2021 Sep 28;16(9):e0257254. doi: 10.1371/journal.pone.0257254 (PMC8478202; doi:10.1371/journal.pone.0257254)

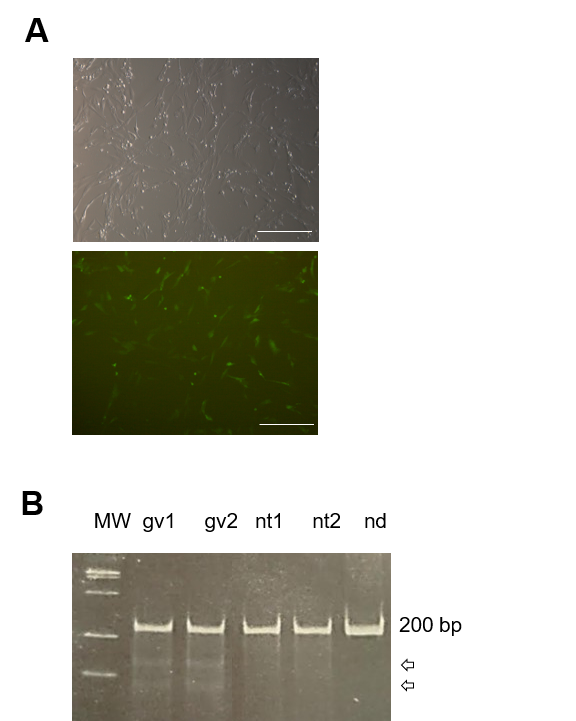


**S1 Figure**

Supplement: S1 Fig — (A) Screening by GFP fluorescent microscopy analysis. Representative image of hFOB transfected with the pSpCas9(BB)-2A-GFP vector. Bright field (upper panel) and fluorescent (lower panel) images are shown. (B) Screening by T7 endonuclease assay. Representative gels indicating, in cells targeted with the guide gRNA-2, the presence of the amplicon fragments (arrow) after T7 endonuclease digestion. About 60–70% transfection efficiency and target specificity were demonstrated. MW: molecular weight; gv: digested amplicon from cells transfected with gRNA containing construct; nt: digested amplicon from not transfected cells; nd: not digested amplicon. (DOCX) [file pone.0257254.s003.docx]

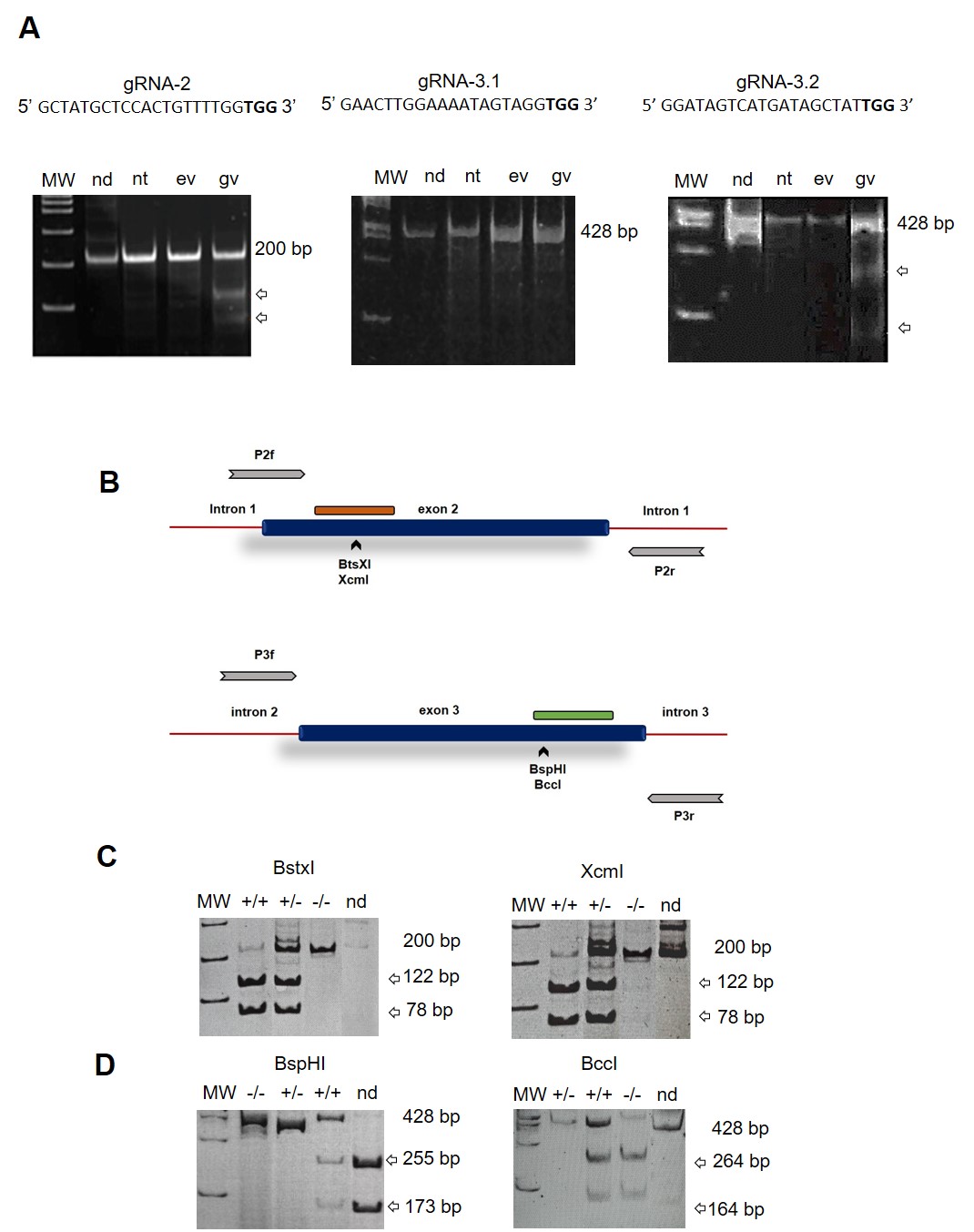


**S2 Figure**

Supplement: S2 Fig — (A) The sequences of the gRNAs are indicated on top of the gels, the PAM sequences are in bold. Representative gels indicating the presence of the amplicon fragments (arrow) after T7 endonuclease digestion in cells targeted with gRNA-2 and gRNA-3.2. MW: molecular weight; nd: not digested amplicon, nt: digested amplicon from not transfected cells, ev: digested amplicon from cells transfected with empty vector; gv: digested amplicon from cells transfected with gRNA containing construct. (B) Scheme of the specific restriction enzymes used to discriminate among targeted and WT clones. Two restriction endonucleases recognizing the WT sequence in the region of the Cas9 cleavage were chosen for each guide to optimize the detection of the mutations inserted by non-homologous-end-join repair system. BtsXI and XcmI digestions were performed for gRNA-2 (in orange), BspHI and BccI digestion for gRNA-3.2 (in green). (C) Clonal lines screening for gRNA-2 targeting. Representative gels showing the bands after BstXI and XcmI digestions of the amplicon obtained from gRNA-2 transfected clones. Arrows indicate the bands upon enzymes cleavage. MW: molecular weight; nd: not digested amplicon. (D) Clonal lines screening for gRNA-3.2 targeting. Representative gels showing the bands after BspHI and BccI digestions of amplicon obtained from gRNA-3.2 transfected clones. Arrows indicate the bands upon enzymes cleavage. MW: molecular weight; nd: not digested amplicon. (DOCX) [file pone.0257254.s004.docx]

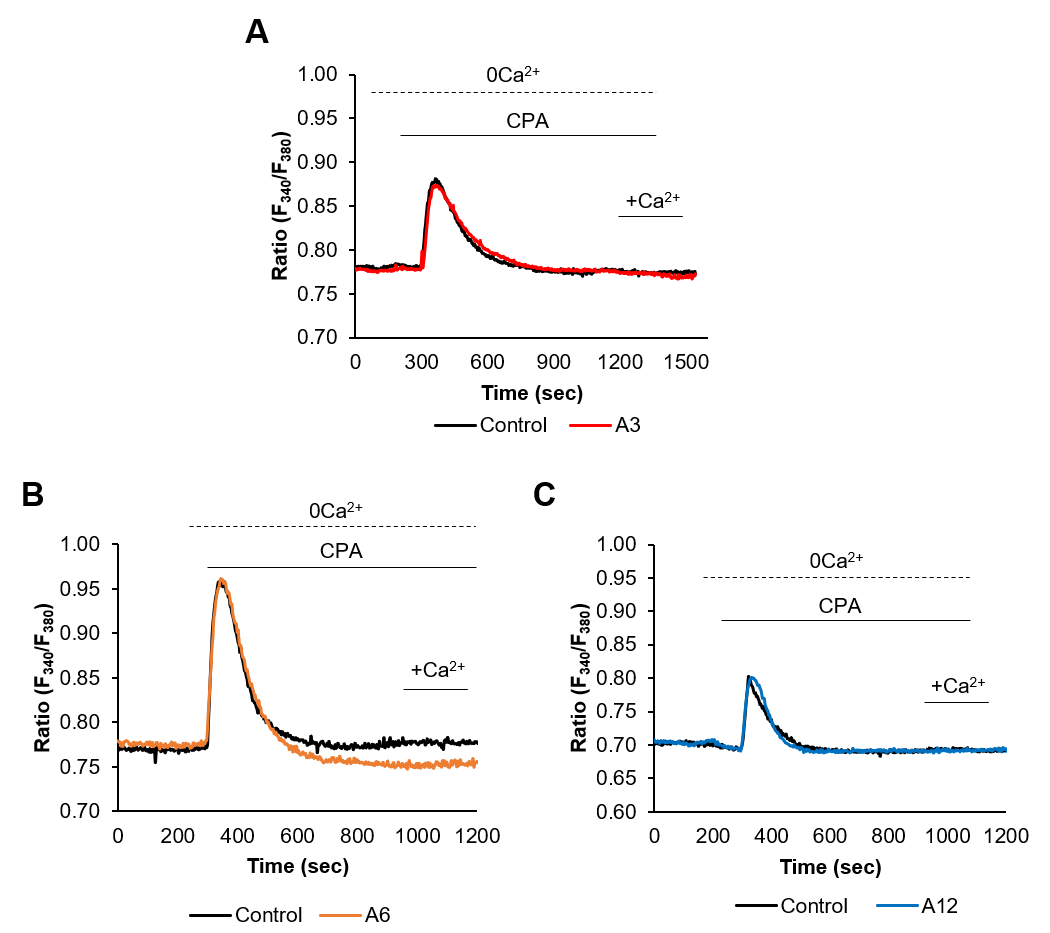


**S4 Figure**

Supplement: S3 Fig — (A) Western blot of TRIC-B and β-Actin of the generated clones. (B) Western blot of ITPR1, 2, 3 isoforms on A3 and control clones with relative total protein staining. (C) Western blot of Collagen type I on A3 and control clones with relative total protein staining. (D) Western blot of Osterix on A3 and control clones with relative total protein staining. (DOCX) [file pone.0257254.s005.docx]

**
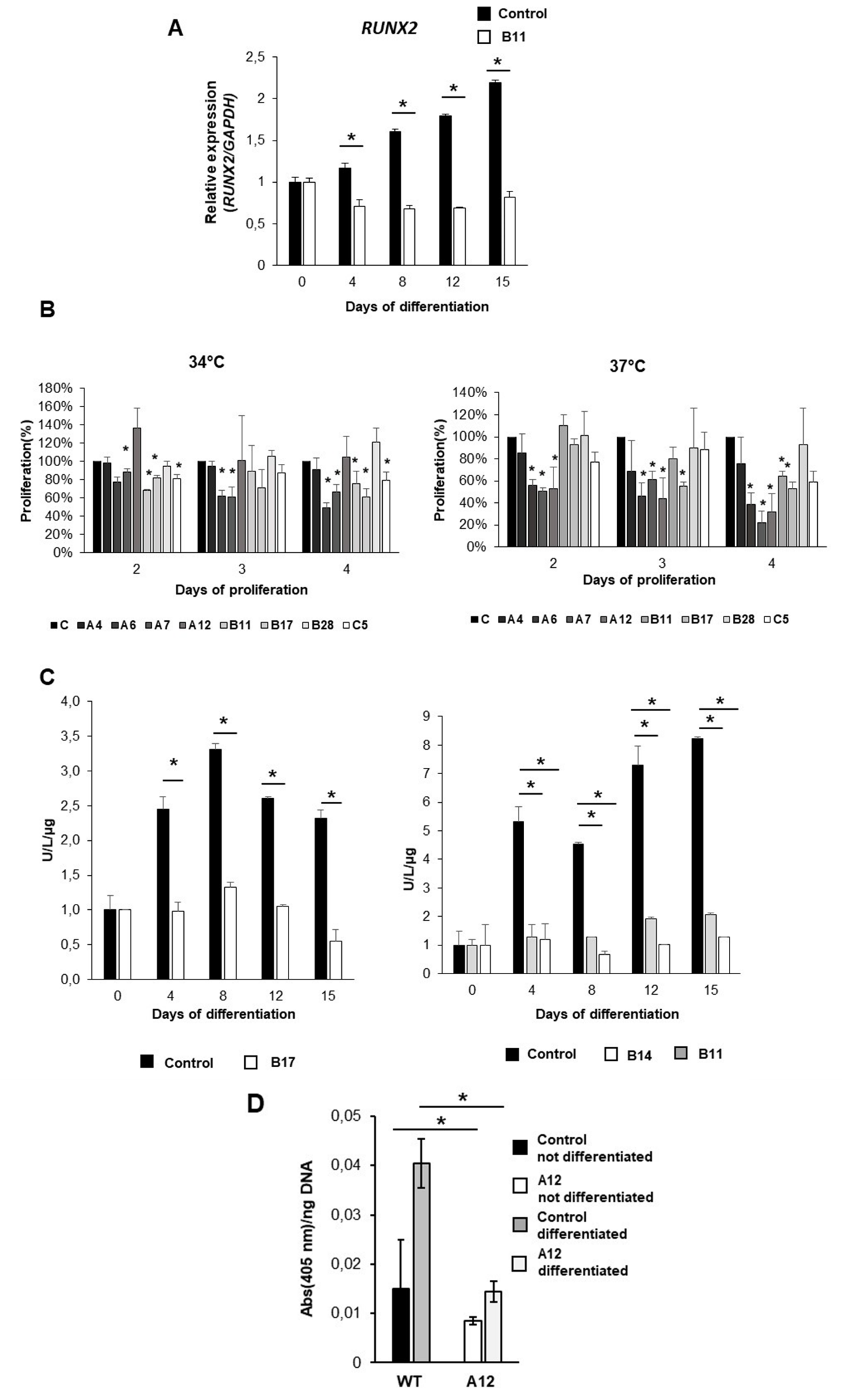
S5 Figure**

Supplement: S4 Fig — (A, B, C) Endogenous Ca2+ release induced by the IP3-producing autacoid UTP (100 μM) was abrogated by depletion of the ER Ca2+ store with cyclopiazonic acid (CPA; 20 μM). (DOCX) [file pone.0257254.s006.docx]

**
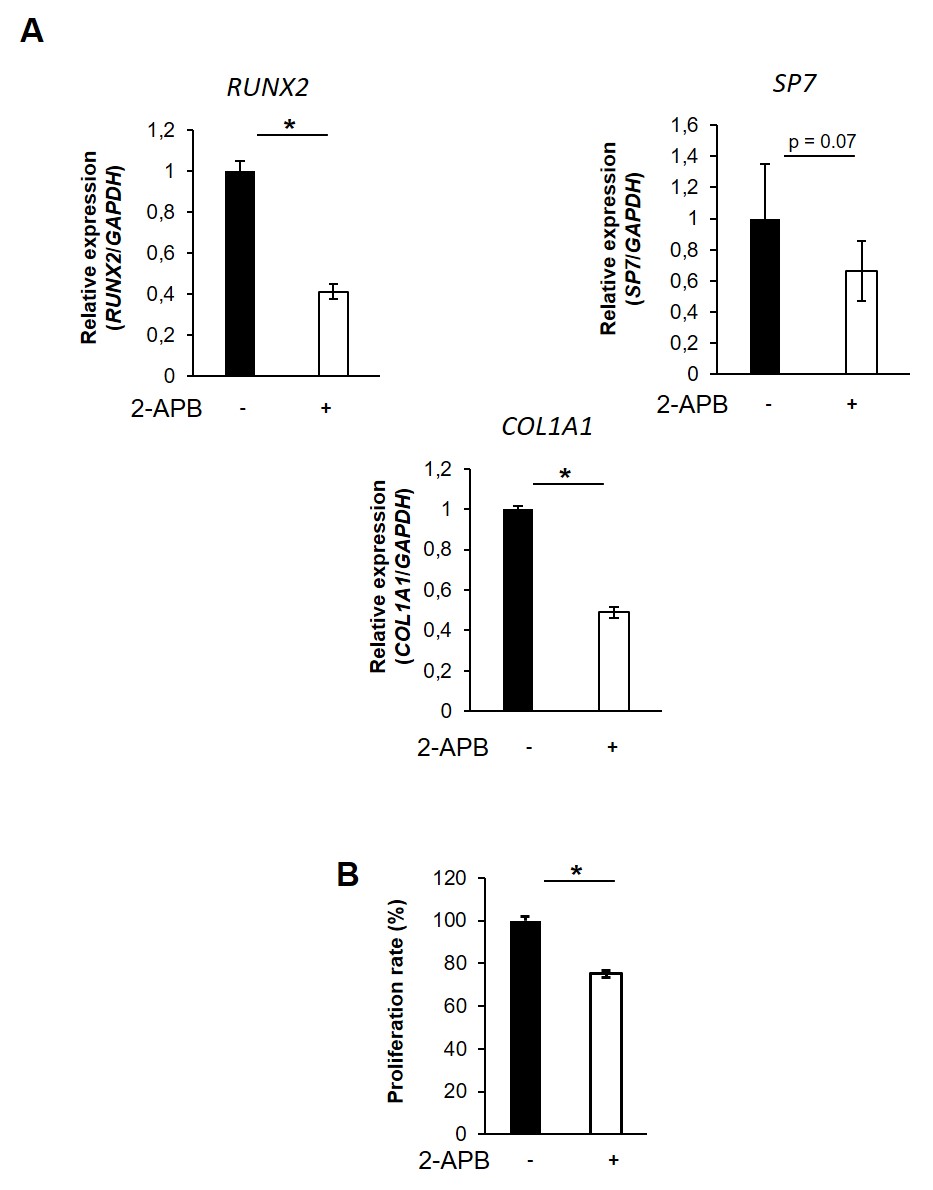
**

**S6 Figure**

Supplement: S5 Fig — (A) Expression analysis of the osteoblastogenic marker RUNX2. RUNX2 expression was reduced in B17 mutant clone with respect to control cells at all time points analysed during hFOB differentiation. (B) Osteoblasts’ proliferation analyses. Proliferation was reduced in the majority of the mutant clones analysed. (C) Alkaline phosphatase (ALP) activity analysis. The activity of ALP was significantly reduced in B17, B11 and B14 mutant clones compared to WT at 4, 8, 12 and 15 days of differentiation. (D) Mineralization level by ARS staining. The mineral amount was significantly reduced in the KO clone A12 with respect to WT in both differentiated and not differentiated conditions. *p<0.05. (DOCX) [file pone.0257254.s007.docx]

**A**

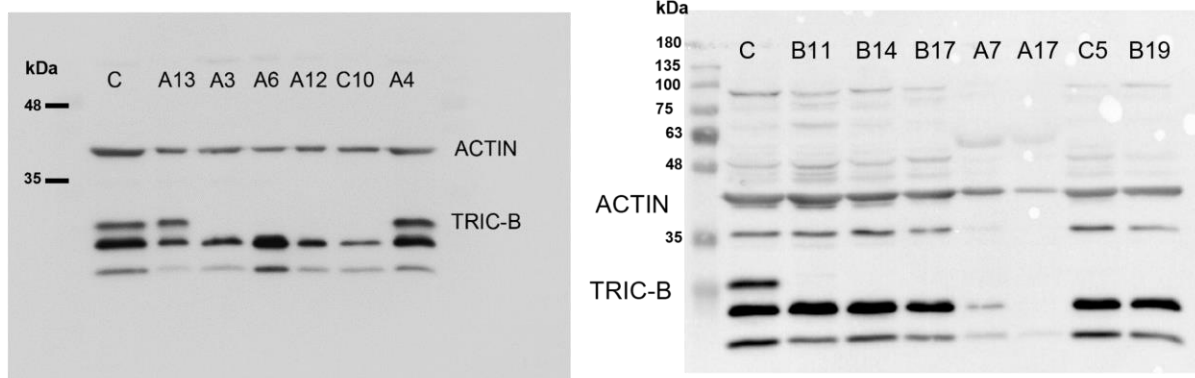

**B**

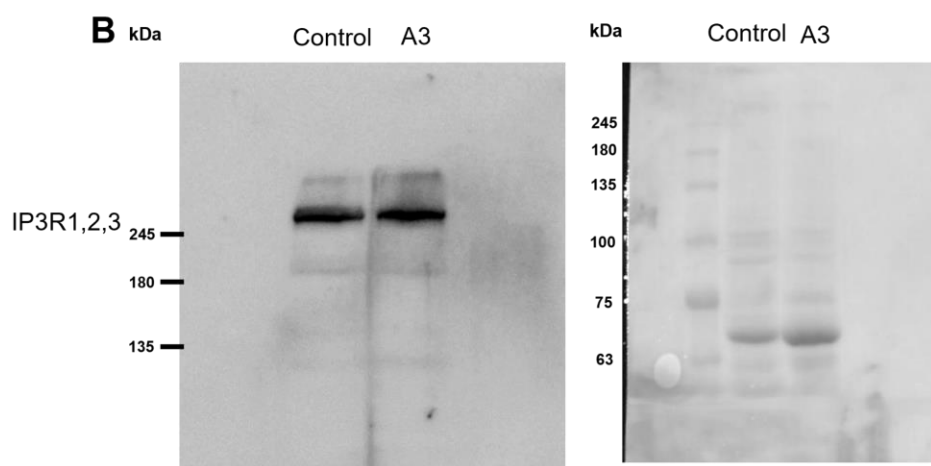

**C**

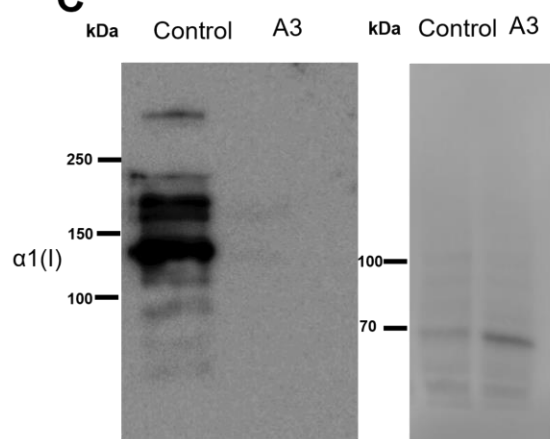

**D**

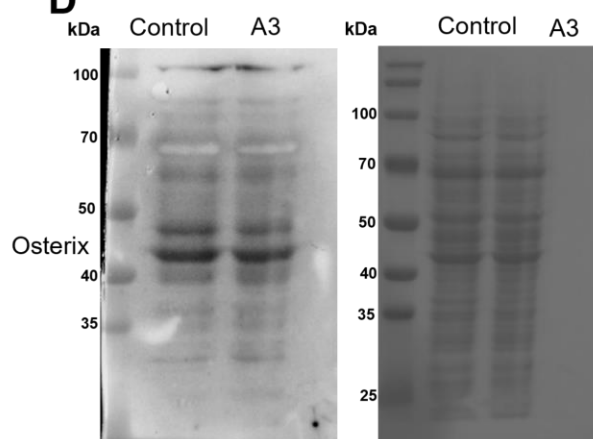

Supplement: S1 Raw images — (PDF) [file pone.0257254.s012.pdf]
