# Supplementary material for: Knocking out TMEM38B in human foetal osteoblasts hFOB 1.19 by CRISPR/Cas9: A model for recessive OI type XIV
Source: PLoS One. 2021 Sep 28;16(9):e0257254. doi: 10.1371/journal.pone.0257254 (PMC8478202; doi:10.1371/journal.pone.0257254)
Supplement: S1 Table — (DOCX) [file pone.0257254.s009.docx]

**S1 Table**. Enzyme digestion for clones transfected with gRNA-2

|  |  | **Enzymes** | |
| --- | --- | --- | --- |
| **Clone** | **Guide** | **BStXI** | **XcmI** |
| A1 | gRNA-2 | Heterozygous | Homozygous |
| A2 | gRNA-2 | Heterozygous | Heterozygous |
| A3 | gRNA-2 | Homozygous | Homozygous |
| A4 | gRNA-2 | Wild type | Wild type |
| A5 | gRNA-2 | Homozygous | Homozygous |
| A6 | gRNA-2 | Homozygous | Homozygous |
| A7 | gRNA-2 | Homozygous | Homozygous |
| A8 | gRNA-2 | Heterozygous | Heterozygous |
| A9 | gRNA-2 | Heterozygous | Heterozygous |
| A10 | gRNA-2 | Heterozygous | Heterozygous |
| A11 | gRNA-2 | Homozygous | Homozygous |
| A12 | gRNA-2 | Homozygous | Homozygous |
| A13 | gRNA-2 | Heterozygous | Heterozygous |
| A14 | gRNA-2 | Homozygous | Homozygous |
| A17 | gRNA-2 | Homozygous | Homozygous |
| A18 | gRNA-2 | Heterozygous | Homozygous |
| A19 | gRNA-2 | Wild type | Wild type |
