# Supplementary material for: Knocking out TMEM38B in human foetal osteoblasts hFOB 1.19 by CRISPR/Cas9: A model for recessive OI type XIV
Source: PLoS One. 2021 Sep 28;16(9):e0257254. doi: 10.1371/journal.pone.0257254 (PMC8478202; doi:10.1371/journal.pone.0257254)
Supplement: S2 Table — (DOCX) [file pone.0257254.s010.docx]

**S2 Table**. Enzyme digestion for clones transfected with gRNA-3.2

|  |  | **Enzymes** | |
| --- | --- | --- | --- |
| **Clone** | **Guide** | **BspHI** | **BccI** |
| B1 | gRNA-3.2 | Heterozygous | Na |
| B2 | gRNA-3.2 | Heterozygous | Heterozygous |
| B3 | gRNA-3.2 | Na | Na |
| B4 | gRNA-3.2 | Heterozygous | Heterozygous |
| B5 | gRNA-3.2 | Heterozygous | Wild type |
| B6 | gRNA-3.2 | Heterozygous | Heterozygous |
| B7 | gRNA-3.2 | Heterozygous | Heterozygous |
| B8 | gRNA-3.2 | Wild type | Heterozygous |
| B9 | gRNA-3.2 | Heterozygous | Heterozygous |
| B10 | gRNA-3.2 | Heterozygous | Heterozygous |
| B11 | gRNA-3.2 | Heterozygous | Heterozygous |
| B12 | gRNA-3.2 | Na | Na |
| B13 | gRNA-3.2 | Wild type | Heterozygous |
| B14 | gRNA-3.2 | Heterozygous | Heterozygous |
| B15 | gRNA-3.2 | Heterozygous | Wild type |
| B16 | gRNA-3.2 | Na | Na |
| B17 | gRNA-3.2 | Heterozygous | Heterozygous |
| B18 | gRNA-3.2 | Heterozygous | Heterozygous |
| B19 | gRNA-3.2 | Heterozygous | Heterozygous |
| B20 | gRNA-3.2 | Heterozygous | Heterozygous |
| B21 | gRNA-3.2 | Heterozygous | Heterozygous |
| B22 | gRNA-3.2 | Wild type | Heterozygous |
| B23 | gRNA-3.2 | Heterozygous | Heterozygous |
| B24 | gRNA-3.2 | Homozygous | Heterozygous |
| B25 | gRNA-3.2 | Na | Na |
| B26 | gRNA-3.2 | Na | Na |
| B27 | gRNA-3.2 | Heterozygous | Heterozygous |
| B28 | gRNA-3.2 | Wild type | Wild type |
| B29 | gRNA-3.2 | Heterozygous | Heterozygous |
| B30 | gRNA-3.2 | Na | Na |
| B31 | gRNA-3.2 | Wild type | Wild type |
| B32 | gRNA-3.2 | Wild type | Heterozygous |
| B33 | gRNA-3.2 | Wild type | Heterozygous |
| B34 | gRNA-3.2 | Wild type | Na |
| B35 | gRNA-3.2 | Heterozygous | Heterozygous |
| B36 | gRNA-3.2 | Heterozygous | Heterozygous |

Na: Not analyzed
