# Supplementary material for: Knocking out TMEM38B in human foetal osteoblasts hFOB 1.19 by CRISPR/Cas9: A model for recessive OI type XIV
Source: PLoS One. 2021 Sep 28;16(9):e0257254. doi: 10.1371/journal.pone.0257254 (PMC8478202; doi:10.1371/journal.pone.0257254)
Supplement: S3 Table — (DOCX) [file pone.0257254.s011.docx]

**S3 Table**. Enzyme digestion for clones transfected with both gRNA-2 and gRNA-3.2.

|  |  | **Enzymes** | | | |
| --- | --- | --- | --- | --- | --- |
| **Clone** | **Guide** | **BStXI** | **XCmI** | **BspHI** | **BccI** |
| C1 | gRNA-2 + gRNA-3.2 | Heterozygous | Heterozygous | Heterozygous | Na |
| C2 | gRNA-2 + gRNA-3.2 | Heterozygous | Heterozygous | Heterozygous | Na |
| C3 | gRNA-2 + gRNA-3.2 | Heterozygous | Heterozygous | Heterozygous | Na |
| C4 | gRNA-2 + gRNA-3.2 | Heterozygous | Heterozygous | Heterozygous | Na |
| C5 | gRNA-2 + gRNA-3.2 | Homozygous | Homozygous | Heterozygous | Na |
| C6 | gRNA-2 + gRNA-3.2 | Wild type | Wild type | Na | Na |
| C7 | gRNA-2 + gRNA-3.2 | Wild type | Heterozygous | Wild type | Na |
| C8 | gRNA-2 + gRNA-3.2 | Homozygous | Heterozygous | Homozygous | Heterozygous |
| C9 | gRNA-2 + gRNA-3.2 | Wild type | Heterozygous | Wild type | Na |
| C10 | gRNA-2 + gRNA-3.2 | Homozygous | Homozygous | Na | Homozygous |

Na: Not analyzed
